# Impact of rapid on-site evaluation combined with endobronchial ultrasound and virtual bronchoscopic navigation in diagnosing peripheral lung lesions

**DOI:** 10.1186/s12890-022-01917-z

**Published:** 2022-03-31

**Authors:** Jia-Chao Qi, Liping Liao, Zhiwei Zhao, HuiXue Zeng, Tiezhu Wang, Miaofen Hu, LiJv Wang, Zhi Wu, Yuming Ye, Yangwu Ou, Zhiming Cai, Qiyin Wu, Qiaozhen Xu, Weiliang Zhang, Wensen Huang, Hao Li, Li Lin

**Affiliations:** 1 Department of Respiratory and Critical Care Medicine, Zhangzhou Affiliated Hospital of Fujian Medical University, No. 59, Shengli Rd., Xiangcheng, Zhangzhou, 363000 Fujian Province People’s Republic of China; 2 Department of Ultrasonic Medicine, Zhangzhou Affiliated Hospital of Fujian Medical University, No. 59, Shengli Rd., Xiangcheng, Zhangzhou, 363000 Fujian Province People’s Republic of China; 3 Department of Otolaryngology, Zhangzhou Affiliated Hospital of Fujian Medical University, No. 59, Shengli Rd., Xiangcheng, Zhangzhou, 363000 Fujian Province People’s Republic of China; 4 Department of Pathology, Zhangzhou Affiliated Hospital of Fujian Medical University, No. 59, Shengli Rd., Xiangcheng, Zhangzhou, 363000 Fujian Province People’s Republic of China

**Keywords:** Rapid on-site evaluation, Ultrasound bronchoscopy, Diagnostic yield, Lung biopsy, Peripheral pulmonary lesions

## Abstract

**Background:**

To investigate the value of endobronchial ultrasound (EBUS) and virtual bronchoscopic navigation (VBN) combined with rapid on-site evaluation (ROSE) in diagnosing peripheral pulmonary lesions (PPLs).

**Methods:**

Between January 1st 2019 to September 1st 2021, EBUS and VBN examination were performed in expected consecutive patients with PPLs who were admitted to Zhangzhou Affiliated Hospital of Fujian Medical University (Fujian, China). Finally, based on the calculation of expected diagnostic yield of R-EBUS biopsy and drop out, 198 eligible patients were randomly divided into ROSE group (100 cases) and non-ROSE group (98 cases). The diagnostic yield of brushing and biopsy, the complications, the procedure time, the diagnosis time and expense during diagnosis were analyzed.

**Results:**

In the ROSE group, the positive rate of EBUS brushing and biopsy were 68%, 84%, respectively. The average procedure time and diagnosis time were 18.6 ± 6.8 min, 3.84 ± 4.28 days, respectively, and the average expense was 643.44 ± 706.56 US.$ (4093.15 ± 4494.67 yuan ¥). In the controls, the positive rate of brushing and biopsy were 44%, 74%, respectively. The average procedure time and diagnosis time were 15.4 ± 5.7 min, 6.46 ± 3.66 days, respectively. And the average expense during diagnosis was 1009.27 ± 713.89 US.$ (6420.28 ± 4541.33 yuan ¥). There was significant difference in the positive rate of EBUS brushing and biopsy, diagnosis time and expense during diagnosis between both groups. And no significant difference was observed in the complications and the procedure time. Additionally, the impact of ROSE on diagnostic yield in right upper lobe and the size of lesion ≤ 2 cm in diameter was significant.

**Conclusion:**

In combination with ROSE, EBUS could significantly improve the positive rate of diagnosing PPLs, shorten diagnosis time and reduce expense during diagnosis. ROSE will be of great importance in the diagnosis of PPLs and medical resource.

## Background

Peripheral pulmonary lesions (PPLs) refer to lesions that are located in the subsegmental bronchi and cannot be visualized directly by bronchoscopy [1]. With the widespread application of computer tomography (CT), PPLs have been increasingly detected [2]. It is becoming increasingly apparent that transbronchial biopsy (TBB) has become an important method for obtaining specimen from PPLs, however, the diagnostic yield widely ranges from 36 to 76% [2–5]. The diagnosis of PPLs is still difficult because of its anatomic location far from segmental bronchus, which is unable to reach the lesion by routine bronchoscopy [3]. Particularly, pathological diagnosis was clinically important for those patients with benign PPLs including tuberculosis and pulmonary fungal diseases that can be distinguished from malignant lesions, which could avoid unnecessary operations and reduce medical expenses. Indeed, lung cancer as the most common PPLs is the leading cause of cancer-related death worldwide [4]. In recent years, radial endobronchial ultrasound (R-EBUS) emerged as a powerful tool during TBB and brushing in PPLs, and it led to the improvement of the diagnostic yield [5].

On the other hand, rapid on-site evaluation (ROSE) introduced by Park could help to quickly evaluate the satisfied specimen, form a preliminary diagnosis and guide the TBB operation in real time [6]. An emerging body of evidence indicates that the use of ROSE during endobronchial ultrasound-guided transbronchial needle aspiration (EBUS-TBNA) is a minimally invasive and highly accurate modality for the diagnosis of lymph node metastasis in lung cancer [7, 8]. Based on ROSE, EBUS-TBNA significantly reduced the number of needle passes and complication rates [9], which can contribute to cost savings in the medical system [10]. Actually, a high concordance rate was reported between ROSE and histologic diagnosis [11].

However, little was known about whether the utility of ROSE can affect the diagnostic yield of EBUS TBB in PPLs [12–14]. Subject to the relatively small size of sample [12] and the cohort design [13, 14], so the inference should be interpreted with caution. Additionally, the current data regarding whether several factors such as location and size of lesion [15, 16], inadequate specimen collection affecting the diagnostic yields of EBUS TBB in PPLs [17] remain confirmed.

The aim of this study was to evaluate the value of R-EBUS in combination with ROSE in diagnosing PPLs and explore factors that can influence the diagnostic yields.

## Methods

### Participants

This was a prospective randomized, controlled trial (RCT). Between January 1st 2019 to September 1st 2021, EBUS and virtual bronchoscopic navigation (VBN) examination were performed in expected consecutive patients with PPLs who were admitted to Zhangzhou Affiliated Hospital of Fujian Medical University (Fujian, China). According to the endpoint designed before we started the study, based on the expected diagnostic yield of R-EBUS biopsy in ROSE group and non-ROSE group reported previously [13, 18] and a potential dropout rate of 5%, we performed the PASS version 21.0 to calculate the suitable sample size. Finally, 198 eligible patients were randomly divided into ROSE group (100 cases) and non-ROSE group (98 cases) based on a web-based randomization system (https://www.jq22.com/webqd4314).

. PPLs were defined as lesions surrounded by pulmonary parenchyma and endoscopically invisible (no evidence of endobronchial lesion, extrinsic compression, submucosal tumor, narrowing, inflammation, or bleeding of the bronchus) [19]. Inclusion criteria: chest CT revealed PPLs which was less than or equal to 3 cm in diameter. Exclusion criteria: severe emphysema, multiple or single bullae in lung parenchyma near to pulmonary lesions, cardiac or pulmonary function insufficiency, hemorrhagic diseases or coagulation disorders, and mental disorder, pregnant women, unable to stop anti-platelet aggregation drugs, patients with advanced peripheral lung cancer and other conditions that cannot cooperate with bronchoscopy.

The study was approved by the ethical committee of Zhangzhou Affiliated Hospital of Fujian Medical University (ethics approval no. Zzsyy-2017-1116), and all patients provided informed written consent.

### Biopsy procedure by EBUS and VBN

The location of the bronchus leading to the lesion was designed by VBN in advance (Ziostation2; Ziosoft Ltd, Tokyo, Japan; LungPoint; Bronchus Ltd, Mountain View, CA, USA; or DirectPath, Olympus Ltd, Tokyo, Japan). Bronchoscopy was performed applying a fiberoptic bronchoscope (BF-260, Olympus, Japan) in combination with the R-EBUS (20 MHz mechanical-radial type, UM-S20-20R or UM-S20-17S; Olympus, Japan) and guide sheath (GS) kit (K-201 or K-203; Olympus, Japan). The scope was inserted through the oral route, and each procedure was performed under local anesthesia with intravenous administration of midazolam for mild sedation. X-ray fluoroscopy (VersiFlex VISTA, Hitachi, Japan) was applied to guide the insertion of the R-EBUS probe with GS through the working channel of the bronchoscope until the target site was reached.

After determining the location of the R-EBUS probe and GS within a target lesion, brushing and TBB cytology were performed for specimen collection. When the R-EBUS probe was adjacent to or outside the target lesion, the bronchus closest to the PPLs was meticulously searched under fluoroscopy prior to collecting specimen. X-ray fluoroscopy guidance was applied during biopsy and brushing sampling, as well as during removal of the GS after sampling. The number of R-EBUS attempts per lesion is less than 5 times and the target value for the number of biopsies is less than 3 times [20]. The procedure time was measured based on the interval between insertion and removal of the bronchoscope through the vocal cords. The diagnosis time was measured based on the interval between the R-EBUS operation to final pathological diagnosis. And the expense during diagnosis indicated the costs between the R-EBUS operation to final pathological diagnosis.

### Rapid on-site specimen evaluation

The material obtained from bronchoscopic biopsy or brushing, was immediately expressed onto numbered glass slides. Diagnostic ROSE specimens were characterised as those clearly demonstrating the typical cytological features of malignancy and inflammation. Non-diagnostic specimens were those where ROSE failed to convincingly demonstrate these features, including where specimens demonstrated only the appearance of benign epithelial cells or where specimens demonstrated a paucity of malignant cells (e.g. < 10 groups of ≥ 5 cells). And bronchoscopy was terminated if ROSE demonstrated diagnostic material. Non-diagnostic rapid on-site examination resulted in further bronchoscopic sampling. What is universally noted is that maximal yield is achieved by a combination of techniques [21, 22]. In agreement with previous study [14], for those unsatisfied or non-diagnostic sample with brushing, especially for those deep lesions, further biopsy specimens were repeatedly rolled on the slide to obtain bronchial mucosal cells and performed with ROSE. It is necessary to performed ROSE with a biopsy sample, which is an important increment in diagnostic yield.

Diff stain was applied for specimen staining (Diff-Quik; Sysmex Ltd. Kobe, Japan). The stained slide was screened by the same experienced cytopathologist, who continuously reported the findings in real time and announced when sufficient diagnostic material had been obtained for a provisional diagnosis. The bronchoscopist modified or terminated the sampling process according to the information provided by the cytopathologist. Then tissue biopsy samples were placed in 10% formalin and were embedded in paraffin for routine histologic evaluation on hematoxylin and eosin staining.

Positive diagnostic criteria for ROSE: (1) The ROSE cytology showed cancer cells and nuclear heterogeneous cells; (2) The ROSE reported neutrophils, and lesions absorbed after anti-infective treatment; (3) The ROSE showed lymphocytes and other inflammatory cells.

For those non-diagnostic patients, the final diagnosis was determined through additional medical examinations such as CT-guided percutaneous lung biopsy, surgical biopsy or anti-infection, anti-tuberculosis therapy and follow-up for at least 6 months.

### Evaluation of complication

Complications are considered as follows: severe bleeding (bleeding volume > 50 ml), pneumothorax, malignant arrhythmia, lidocaine poisoning, monitoring blood oxygen saturation < 90%, blood pressure greater than 180/120 mmHg or less than 90/60 mmHg, consciousness disorder or other adverse events.

### Endpoint and statistical analysis

The Primary outcome was the diagnostic yield of R-EBUS brushing and biopsy. Second outcome was bronchoscopy complications, the procedure time and diagnosis time. Before starting the study, we set the expected diagnostic yield of R-EBUS biopsy in ROSE group and a potential dropout rate based on previous reports. And we used the PASS version 21.0 to calculate the expected sample size. SPSS version 20.0 (SPSS, Inc., Armonk, NY, USA) was applied for statistical analyses. All variables were evaluated for normal distribution prior to analysis. Non-normally distributed continuous variables were expressed as the median (Md) and interquartile range (IQR), using Kruskal–Wallis H (K) for multiple-group comparison. Normally distributed data were expressed as mean ± SD, using a Student’s *t* test or one-way ANOVA for comparison. Each nodule attempt was characterized as successful or unsuccessful, based on the definitions provided above for the primary and secondary outcomes. Categorical variables including study outcomes were presented as number (percentage). A comparison of study outcomes between ROSE group and non-ROSE group was done using the chi-square test or Fisher’s exact test. Differences were considered to indicate significance if a *p* value was < 0.05.

## Result

### Clinical characteristics of patients

Table 1 shows that there are 198 subjects enrolled in this study, including 116 male patients and 82 female patients; the average diameter of the lesions in the ROSE group is 2.84 ± 2.28 cm, and the control group is 2.48 ± 2.66 cm. There were 87 cases of lung tumors and 7 cases of pulmonary tuberculosis in the ROSE group, 83 cases of lung tumors and 8 cases of pulmonary tuberculosis in the non-ROSE group. No significant difference in lesion size, location of lesion and composition of disease was observed between both groups.

**Table 1:** analysis of clinical characteristics in the study

Characteristics	ROSE Group	Non-ROSE Group	*p* value
n	100	98	
Age (year)	45.23 ± 10.34	46.17 ± 12.24	0.675
Sex (male/female)	59/40	57/42	0.847
Diameter (cm)	2.84 ± 2.28	2.48 ± 2.66	0.801
*Location of lesion*			
Right upper lobe	18 (18%)	21 (21.4%)	0.623
Right middle lobe	16 (16%)	15 (15.3%)	0.808
Right lower lobe	24 (24%)	22 (22.4%)	0.821
Left upper lobe	22 (22%)	18 (18.7%)	0.693
Left lower lobe	20 (20%)	22 (22.4%)	0.721
*Final pathological diagnosis*			
Adenocarcinoma	75 (75%)	74 (75.5%)	0.808
Squamous carcinoma	5 (5%)	4 (4.1%)	0.723
NSCLC, not otherwise specified	2 (2%)	2 (2.1%)	0.921
Carcinoid	2 (2%)	2 (2.1%)	0.921
Metastatic malignancy	3 (3%)	1 (1.0%)	0.621
*Benign*			
Tuberculosis	7 (7%)	8 (8.2%)	0.813
Pulmonary aspergillosis	4 (4%)	5 (5.1%)	0.723
Others	2 (2%)	2 (2.1%)	0.921

Normally distributed data were expressed as mean ± SD, using a Student’s *t* test for comparison. Categorical variables were presented as number (percentage), using the chi-square test or Fisher’s exact test when compared. Differences were considered to indicate significance if a *p* value was < 0.05

ROSE, rapid on-site evaluation; NSCLC, non small cell lung cancer

### The impact of ROSE on the diagnostic yield of R-EBUS brushing and biopsy

Table 2 indicates that the positive rate of brushing in the ROSE group is 68%, and the control group is 44%. The positive rate of biopsy in the ROSE group is 84%, and the controls is 74%. The differences in the diagnostic yield of R-EBUS brushing and biopsy between both groups were significant.

**Table 2:** Difference in the diagnostic yield of R-EBUS brushing and biopsy in PPLs between both groups

	ROSE Group	Non-ROSE Group	*X*^2^	*p* value
Diagnostic yield of brushing	68/100 (68%)	44/98 (44.9%)	19.05	0.000
Diagnostic yield of biopsy	84/100 (84%)	75/98 (75.5%)	8.7	0.001

Categorical variables were presented as number (percentage), using the chi-square test or Fisher’s exact test when compared. Differences were considered to indicate significance if a *p* value was < 0.05

ROSE, rapid on-site evaluation; PPLs, peripheral lung lesions; R-EBUS, radial endobronchial ultrasound

### The impact of ROSE on bronchoscopy complications

Table 3 shows 2 cases of severe bleeding in ROSE group, and there is no significant difference in the incidence of bronchoscopy complications between both groups.

**Table 3:** Differerce in the incidence of bronchoscopy complications between both groups

	ROSE Group	Non-ROSE Group	*X*^2^	*p* value
n	100	98		
Positive cases	2	0		
Incidence	2%	0%	0.990	0.320

Categorical variables were presented as number (percentage), using the chi-square test or Fisher’s exact test when compared. Differences were considered to indicate significance if a *p* value was < 0.05

ROSE, rapid on-site evaluation

### The impact of ROSE on the procedure time and diagnosis time

Table 4 shows that the average procedure time in the ROSE group is 18.6 ± 6.8 min, and the control group is 15.4 ± 5.7 min. There was no significant difference in both groups. And the average diagnosis time in the ROSE group was 3.84 ± 4.28 days, and the controls was 6.46 ± 3.66 days. Significant difference was observed in both groups.

**Table 4:** Difference in the procedure time and diagnosis time between both groups

	ROSE Group	Non-ROSE Group	*p* value
n	100	98	
Procedure time(min)	18.6 ± 6.8	15.4 ± 5.7	0.231
Diagnosis time(days)	3.84 ± 4.28	6.46 ± 3.66	0.001

Normally distributed data were expressed as mean ± SD, using a Student’s *t* test for comparison. Differences were considered to indicate significance if a *p* value was < 0.05

ROSE, rapid on-site evaluation

### The impact of ROSE on the expense during diagnosis

Table 5 shows that the average expense during diagnosis in the ROSE group is 643.44 ± 706.56 US.$ (4093.15 ± 4494.67 yuan ¥), and the control group is 1009.27 ± 713.89 US.$ (6420.28 ± 4541.33 yuan ¥). There was statistically significant difference.

**Table 5:** Difference in the expense during diagnosis between both groups

	ROSE Group	Non-ROSE Group	*p* value
n	100	98	
Expense (US.$)	643.44 ± 706.56	1009.27 ± 713.89	0.011

Normally distributed data were expressed as mean ± SD, using a Student’s *t* test for comparison. Differences were considered to indicate significance if a *p* value was < 0.05. ROSE: rapid on-site evaluation

### The impact of ROSE on diagnostic yield related to location and size of the lesion between both groups

Table 6 shows that the diagnostic yield of TBB in right upper lobe in the ROSE group is significantly higher than the controls. The significant difference was also observed in the size of lesion ≤ 2 cm in diameter.

**Table 6:** Diagnostic yield related to location and size of the lesion between both groups

Variables	Diagnostic yield (%)		*X*^2^	*p* value
	ROSE Group	Non-ROSE Group		
*Location of lesion*				
Right upper lobe	18/20 (90%)	13/22 (59.1%)	0.505	0.047
Right middle lobe	14/16 (87.5%)	14/16 (87.5%)	0.00	1.000
Right lower lobe	20/22 (90.9%)	14/18 (77.7%)	0.636	0.425
Left upper lobe	15/23 (65.2%)	15/25 (60%)	0.221	0.638
Left lower lobe	15/19 (78.9%)	13/17 (76.5%)	0.469	0.493
*Size of lesion* (cm)				
< 2				
Brushing	24/48 (50%)	12/46 (26.1%)	4.117	0.035
Total diagnostic yield	36/48 (75%)	26/46 (56.5%)	0.983	0.042
> 2				
Brushing	44/52 (84.6%)	40/52 (76.9%)	2.782	0.213
Total diagnostic yield	50/52 (96.1%)	48/52 (88.9%)	1.020	0.572

Categorical variables were presented as number (percentage), using the chi-square test or Fisher’s exact test when compared. Differences were considered to indicate significance if a *p* value was < 0.05

ROSE, rapid on-site evaluation

## Discussion

Combined with VBN, the positive rate of EBUS TBB on PPLs less than 2.0 cm in diameter could reach 44% [23]. Our study has confirmed the performance of ROSE that it is likely to improve diagnostic yield especially for size of lesion < 2 cm. In disagreement with previous data suggesting reduced operation time in ROSE group [12], we reported that ROSE could shorten the diagnosis time, and improve cost-effectiveness for R-EBUS patients. On the basis of ROSE findings consistent with subsequent final pathologic diagnosis, it supports our approach of termination of procedure in the event of ROSE revealing malignancy and inflammation without further sampling.

Particularly, ROSE has revealed clinical importance in the diagnosis of lung tumors, lung nodules, mediastinum disease and other diseases [24]. Compared with the controls [25], ROSE could reduce the number of unnecessary punctures by 33%, meanwhile, it could make 68% of patients succeed in one puncture during TBNA. Although there were increasing researches on ROSE with the widespread application of TBNA, unfortunately, reports showed the controversial results [7, 26, 27]. Nakajima and his colleagues were in favor of our findings that most patients with suspected lung cancer could be diagnosed by lung biopsy pathology in combination with ROSE, and the consistency between ROSE and the final pathological diagnosis was 94.3% [26]. Conversely, Griffin et al. found that ROSE did not increase the positive rate of EBUS-TBNA, and it also did not reduce the number of punctures. Besides, ROSE increased expense, labor and time waste [27]. Also, there was no significant difference in diagnostic sensitivity and accuracy between ROSE group and the controls [7]. And it is now accepted that that ROSE could not increase the diagnostic yield of TBNA or EBUS-TBNA for skilled operators [28].

To the best of our knowledge, limited data are available concerning the application of ROSE during R-EBUS TBB in the diagnosis of PPLs [12–14, 18]. A prospective RCT enrolling 152 patients with PPLs suggested that ROSE could improve the diagnostic yield and shorten the operation time. Meanwhile, no severe procedure related complications were observed, such as pneumothorax and hemorrhage [12]. The study was clearly in favor of our results, however, we should note that the researchers did not utilize brushing in the R-EBUS procedure, and the difference in the incidence of hemorrhage between both groups was signicant. In agreement with previous studies, we found that the number of unsatisfactory specimens in ROSE group decreased [14, 17], then the diagnostic yield of PPLs based on ROSE especially malignant tumors was improved [23]. However, the study conducted by Steinfort did not include control group, so the limitation may lead to some unnecessary bias [14]. Restricted to the sample size [12] and the cohort design [13, 14], so the conclusions should be discussed with caution.

Our findings demonstrated that ROSE could quickly evaluate whether the samples obtained are satisfactory, form a preliminary diagnosis in real time. Based on the good consistency between ROSE and final diagnosis, the positive result supported termination of procedure without further sampling. It is obvious that EBUS combined with ROSE can reduce the operation time [12, 18]. And the improvement of the efficiency of bronchoscopy by ROSE can reduce the adverse physiological effects to some degree [29] and incidence of second biopsy. Furthermore, ROSE could show several neutrophils or macrophages or tumor cells, then bacterial culture or detailed biological characterization would be recommended [30]. In disagreement with previous study [12], Our data reported no significant difference in the operation time. But the diagnosis time was significantly shorter in ROSE group than the controls. Previous prospective, randomized studies were performed to evaluated the impact of ROSE in terms of cost-effectiveness including the need for further specimen collection and procedure time. Indeed, reduced overall costs were shown in the Utility of ROSE of TBNA [17, 31]. Importantly, we firstly reported the significantly decreased expense based on ROSE in combination with EBUS biopsy. And we speculated that the reduction of expense during diagnosis would be responsible for the reduced cases including severe complications and the chance of second biopsy, which contributed to public health resources greatly [32].

The majority of data suggested that some factors may affect the diagnostic yield of PPLs, such as the size of lesions and the location of lesions and so on [33]. Particularly, we were supported that ROSE can significantly improve the diagnostic yield of the lesions in diameter ≤ 2.0 cm [12]. And it may be attributed to the technical focus and tracheal structure reasons [33], which highlighted the clinical importance of this method in small lesions. On the other hand, supported by Steinfort [14], we observed no association between lesion size and ROSE outcome. Indeed, it should be noted that it was a cohort study and size of lesions fluctuated greatly. Besides, our study was in accordance with previous researches that the diagnostic yield of upper lobe was relatively low [34, 35]. We hypothesized that the bending angle of the upper lobe branch is too large, and the ultrasound probe is unable to stick to the focus and the sampling tool cannot extend to the distant focus according to the path of the probe. Under the guidance of ROSE, the extension path of biopsy forceps was modified to reach the lesion accurately, leading to an improvement of diagnostic yield in difficult cases.

There are still some limitations in our findings. Firstly, although our study was a prospective RCT, it was a single-center design, the conclusion need to be interpreted with caution. Secondly, although we have set the expected diagnostic rate from basic study data and calculated the sample size before start of the study, it should be noted that the expected diagnostic rate from fewer previous studies may lead to some bias [13, 18]. Meanwhile the sample size of our study was relatively small, so it needs to be expanded to reduce unnecessary bias in the future. Thirdly, we applied the Diff-Quik staining in our study, which is a modification of the Wright–Giemsa stain, whereas other researches used modified Shorr stain for slide preparations [36]. Different staining methods were reported to be associated with varying sensitivity [37]. We may need a optimal staining method for ROSE in future studies. Fourthly, it may be a little different in every patients’ examination and treatment before diagnosis, which led to some bias in expense. In spite of this, it could still reflect every patient's cost during diagnosis to a certain degree.

## Conclusions

In combination with ROSE, EBUS could increase the diagnostic yield of PPLs, shorten the diagnosis time, leading to a reduction of expense during diagnosis. ROSE would be of importance in diagnosing PPLs and medical burden.

## Data Availability

The datasets used and/or analyzed during the current study are available from the corresponding author on reasonable request.

## Abbreviations

PPLs: Peripheral pulmonary lesions
CT: Computer tomography
TBB: Transbronchial biopsy
ROSE: Rapid on-site evaluation
R-EBUS: Radial endobronchial ultrasound
RCT: Controlled trial
IQR: Interquartile range
NSCLC: Non small cell lung cancer
TBNA: Transbronchial needle aspiration
VBN: Virtual bronchoscopic navigation
